# Panpipes as units of cultural analysis and dispersal

**DOI:** 10.1017/ehs.2020.15

**Published:** 2020-05-21

**Authors:** Gabriel Aguirre-Fernández, Damián E. Blasi, Marcelo R. Sánchez-Villagra

**Affiliations:** 1 Palaeontological Institute and Museum, University of Zurich, Zurich, Switzerland; 2 Radcliffe Institute for Advanced Study, Harvard University, Cambridge, MA, USA; 3 Department of Linguistic and Cultural Evolution, Max Planck Institute for the Science of Human History, Jena, Thuringia, Germany; 4 Quantitative Linguistics Laboratory, Kazan Federal University, Kazan, Republic of Tatarstan; 5 Institute for the Study of Language Evolution, University of Zurich, Zurich, Switzerland; 6 Human Relations Area Files, Yale University, CT, USA

**Keywords:** Cultural evolution, ethnomusicology, random forests, South America, Melanesia

## Abstract

The panpipe is a musical instrument composed of end-blown tubes of different lengths tied together. They can be traced back to the Neolithic, and they have been found at prehistoric sites in China, Europe and South America. Panpipes display substantial variation in space and time across functional and aesthetic dimensions. Finding similarities in panpipes that belong to distant human groups poses a challenge to cultural evolution: while some have claimed that their relative simplicity speaks for independent inventions, others argue that strong similarities of specific features in panpipes from Asia, Oceania and South America suggest long-distance diffusion events. We examined 20 features of a worldwide sample of 401 panpipes and analysed statistically whether instrument features can successfully be used to determine provenance. The model predictions suggest that panpipes are reliable provenance markers, but we found an unusual classification error in which Melanesian panpipes are predicted as originating in South America. Although this pattern may be signalling a diffusion event, other factors such as convergence and preservation biases may play a role. Our analyses show the potential of cultural evolution research on music that incorporates material evidence, which in this study includes both archaeological and ethnographic samples preserved in museum collections.

## Graphical abstract

**Figure:**
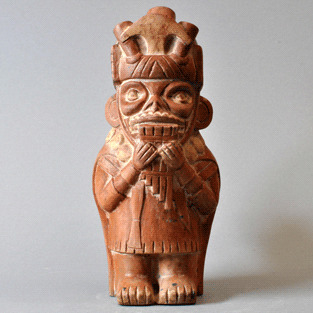

**Media summary:** Panpipe diversity in space and time – the cultural evolution of a musical instrument.

## Introduction

**Figure 1.**
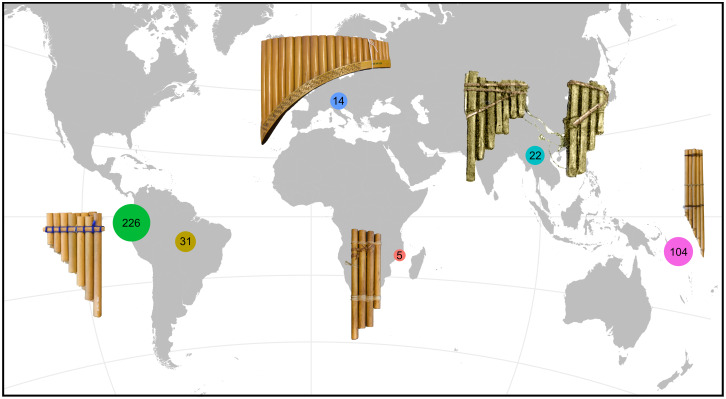
Panpipe sample size according to region. Andes and West coast (green), Amazon and Caribbean region (olive), Europe (blue), Africa (pink), South East Asia (turquoise) and Melanesia (violet).

The term ‘panpipe’ refers to a group of aerophones characterized by having several end-blown tubes of different pitch combined to form a single instrument (Figure 1). These tubes are blown across their upper ends and usually stopped at their lower ends, and tied together forming either a raft or a bundle (Sachs, 1940). Panpipes are found in human groups in Africa, Asia and Europe (Figure 1), but they are most prevalent and deeply embedded in areas of Melanesia and South America (Baines, 1962). Potential iconographic evidence of the panpipe goes back to Anatolia, ca. 8000 BCE (Adorján & Meierott, 2008). The instrument also appeared very early (before 1500 BCE) in China, as attested by pictographs and classic texts (McKinnon et al., 2001). Two bamboo panpipes found in the tomb of Marquis Yi of Zeng (who died in 433 BCE) are exceptionally well preserved and among the oldest direct evidence of panpipes in China (Bagley, 2005). The earliest European representations of panpipes in three bronze urns from Italy are dated 600–400 BCE, later reaching peak popularity among the Etruscans (McKinnon et al., 2001). Panpipes were present and diverse in South America long before contact with Europeans (Pérez de Arce, 1986). Archaeological and ethnographical records indicate a distribution along the Pacific coast from Panama (Guna culture) to Chile (Tolten); their distribution continues eastwards through the Amazon and reaches the Atlantic and Caribbean coasts (Aretz, 1967). The oldest American panpipes are arguably dated 5700 years old and were found in Chilca, Peru (Bolaños, 2007; Mansilla-Vásquez, 2009). Ancient tombs of the Paracas (ca. 800–100 BCE), Nasca (ca. 200 BCE–600 CE), and Moche (ca. 200–900 CE) cultures have yielded panpipes mostly made from ceramic, cane and silver (Olsen, 2002). Panpipes are virtually absent in North America, with the rare exception of the Hopewell culture (200 BCE–500 CE) that produced three- to four-tube (cane) panpipes wrapped in a cooper ‘jacket’ (Cree, 1992; Turff & Carr, 2005; Young, 1970). In Oceania, the panpipes were probably brought by the Lapita (McLean, 2008), becoming predominant in Melanesia and particularly diverse in the Solomon Islands (McLean, 1999); they reached as far east as Tonga, where they are no longer used (Kaeppler, 1974).

The wide geographical and temporal distribution of panpipes is paralleled by a substantial variety in functional structure and aesthetic properties (Supplementary File 1). Panpipes have been made from organic (e.g. reed, bamboo and bones) and inorganic (clay and stone) materials; tube counts can be as few as two, but can also reach large numbers as in the Ecuadorian rondador (more than 40 tubes). Some panpipes are highly ornamented with paintings and/or carvings (e.g. those of the Nasca culture), whereas other lack ornamentation. The smallest panpipes can fit on the palm of a hand, whereas some panpipes in South America and Melanesia can reach lengths greater than a metre. As in the case of other human-made artefacts, e.g. palaeoindian points (O'Brien Darwent, & Lyman, 2001) and Turkmen carpets (Tehrani & Collard, 2002), the history and dynamics of panpipes constitute a case of material cultural evolution. Strong debates on cultural diffusion have traditions in anthropology and archaeology (Campbell, 1960; Kroeber, 1923), but empirical data are strongly needed to advance on specific issues (Jordan, 2014). These current discussions and the availability of analytical methods to study cultural evolution (Leroi & Swire, 2006; Lipo, O'Brien, Collard, & Shennan, 2006) make a test on panpipes timely. Whereas much effort has recently focused on music itself (Savage, 2019), musical instruments have received little attention (but see Tëmkin, 2004; Tëmkin & Eldredge, 2007; Chitwood, 2014).

**Figure 2.**
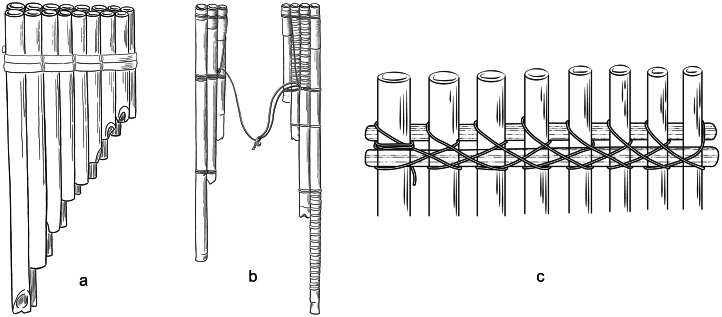
Panpipe features discussed in Sachs (1940): (a) arrangement in two rows; (b) two ‘halves’ of an instrument tied by a lace; and (c) the use of splints to hold the tubes together. A detailed list of characters is provided in Supplementary File 1.

Crucially, contrasting hypotheses have been entertained about the capacity of panpipes to carry and preserve historical signals. On the one hand, panpipes have been suspected to indicate contact events between far-distant human societies that could have taken place thousands of years before present. In particular, specialists have noted that panpipes in South America and Oceania display substantial similarities (e.g. the arrangement in two rows, dual instruments and the use of a cane splint to hold the tubes together; Figure 2), which, in addition to other musical properties between the regions, such as the strong affinity of the absolute pitches and the scales, point to a potential Trans-Pacific contact (Sachs, 1940). Precolumbian contacts have been thoroughly debated (Riley, Kelley, Pennington, & Rands, 1971), but discussions have recently been reignited based on a new wave of analyses and data (Jones, Storey, Matisoo-Smith, & Ramírez-Aliaga, 2011; Lawler, 2010). Examples include the presence of Austronesian genes in some Amazonian Native American societies (Skoglund et al., 2015) and pre-European admixture of Polynesian and South American genes in Rapanui (Moreno-Mayar, Benoit, McKey, & Lebot, 2014), the patterns of diffusion of the sweet potato into Oceania (Roullier et al., 2013) and the introduction of Polynesian chickens into Chile (Storey et al., 2007). These connections are not limited to the exchange of genes or domesticated species: there are parallels in myth cults and gender relations (Thuillard, Le Quellec, d'Huy, & Berezkin, 2018), some reflected in the use and performance of musical instruments, such as the bullroarer (Gregor & Tuzin, 2001). Recent work on linguistics has also conjectured a deep-time link among languages in those regions: features such as the presence of inclusive/exclusive distinctions in pronominal systems, are believed to indicate shared retentions that precede the expansion of humans into the Americas and the Pacific (Bickel, 2015; Bickel & Nichols, 2006; Nichols, 1992). All of these hypotheses are the subjects of ongoing debates (Fehren-Schmitz et al., 2017; Thomson et al., 2014; Matschiner, 2019). Conversely, the apparent simplicity of the panpipe in contrast to other musical instruments has been used to argue for its independent origin. For example, Montagu (2007) pointed out that the wind whistling across the end of a broken reed inspired the making of panpipes according to legends of several cultures. Izikowitz (1935) neglected the diffusionist hypotheses of Sachs (1940; see also Figure 2) because some of those panpipe features also occur in other instruments: combining several tubes into a single instrument also happens in duct whistles; and performance in pairs occurs also in connection with slit drums, trumpets and various flutes. Thus panpipes offer an ideal scenario for strong inference in cultural evolution (Platt, 1964), with competing hypotheses at the opposing ends of the historical signal spectrum. Here we provide the first comprehensive study of panpipe features as cultural units based on the analysis of a sample of 401 panpipes from the world over. We focus on determining whether the properties of these instruments can be leveraged to infer their geographic source at a global level and we discuss why classification errors in such analyses are also informative, providing points of reference for finer-scale studies.

## Methods

### Data preparation

In light of the large diversity exhibited by panpipes, evaluating the potential historical signal carried by the instruments demands a high- and wide-coverage sample of artefacts. Under this premise we analysed 401 instruments covering South America, the Pacific Islands and the Old World, with a time range between today and 3000 years before present (Paracas and Chavín cultures). Those objects with imprecise (e.g. a continent as place of origin) or ambiguous (e.g. place of origin stated with question marks) information were not considered for the analysis. A maximum of 20 features were coded for each instrument, including traits that are structural (e.g. order of tubes) and others that are ornamental (e.g. painted surface). Some materials precluded an overall scoring of instruments (e.g. clay panpipes lacking ligatures) and therefore those characters were scored as non-applicable (NA). Missing values were imputed using the missForest function, a non-parametric method for mixed-type data (Stekhoven & Bühlmann, 2011). The collections visited and the 20 features are detailed in Supplementary File 1. The matrix in csv format is available as Supplementary File 2.

The museum data on instrument provenance are heterogeneous: some museum entries refer to societies or specific villages, but the majority refer to countries. Therefore, for such a comprehensive study, the use of geographic boundaries and roughly defined areas, although suboptimal, is the best way to homogenize the data. We segregated the data into two subsets, which were devised considering the trade-off between information granularity and sample-size homogeneity. ‘Area fine’, used in the first study, includes 11 categories (*n =* instrument sample size): Congo (*n =* 5); Andes (*n =* 225); China (*n =* 12); Europe (*n =* 14); West Papua (*n =* 3); Papua New Guinea (*n =* 20); South America not Andes (mostly Amazonas, but also Caribbean coast; *n =* 31); South East Asia (*n =* 10); Solomons (*n =* 72); Tonga (*n =* 2); Vanuatu (*n =* 7). The second study (‘Area coarse’) includes four categories: Andes (*n =* 225); South America not Andes (*n =* 31); Melanesia (*n =* 104); and Old World (*n =* 40). Further details can be obtained from Supplementary files 2 (matrix) and 3 (code, additional figures).

Missing information is mostly confined to variables related to the material being used to construct the instrument (see also Methods section). Single values of some features are prominently frequent, e.g. almost all panpipes have a stopped lower end and almost all are ‘rafts’. There are some feature values that correlate with others, material being very prominent. Such details on the data structure can be found in Supplementary File S3.

### Exploring structure in panpipes

As a first step we aim at exploring the overall suitability of panpipes as plausible objects carrying faithful information on their provenance. First of all, we determine the number of unique instruments (i.e. unique combination of panpipe traits) and the average dissimilarity among all instruments in our sample. For this purpose we use a simple Gower metric, where the dissimilarity between any two instruments is the fraction of all feature values (that are found in both instruments) that receive a different feature value. In order to calibrate these findings, and given the complex nature of the data at hand (involving important imbalances between classes, missing data and potentially noisy feature assignments), we develop a baseline of comparison. This baseline consists of *N* = 1000 comparable datasets which result from randomly permuting the feature values of each trait in the original data.

We complement these coarse evaluations with a low-dimensional inspection of the whole dataset. As a way of projecting the 20 dimensions of description of the panpipes onto a two-dimensional space, we employ a *t*-distributed stochastic neighbour embedding (*t*-SNE) approach on the set of dissimilarities between panpipes previously discussed. The *t*-SNE is a dimensionality-reduction method that preserves (mostly) local information (i.e. neighbouring data points in the low-dimensional space will tend to reflect adjoining data points in the high dimensional space) (*t*-SNE; van der Maaten & Hinton, 2008). We find this technique more suitable for our purposes than other popular approaches (such as PCA or factor analysis) since we are not interested in overall global dimensions of variation but instead we focus on emerging clusters of instruments displaying non-trivial information about their provenance. The *t*-SNE algorithm has a free parameter – the perplexity – that roughly proxies the number of effective neighbours each observation has. In other words, the larger the perplexity is, the larger we expect clusters of similar panpipes to be. Given the complex nature of the data, it is reasonable to explore a range of plausible values (Wattenberg, Viégas, & Johnson, 2016). We consider perplexities of 2, 5, 10, 25, 50 and 100.

### Panpipe classification through random forests

In order to determine the provenance of panpipes given their features, we trained random forest classifiers (Hothorn & Zeileis, 2015). Since random forests were introduced by Breiman (2001), they have proven useful for many classification tasks (e.g. drug response prediction, identification of DNA proteins, speech and handwriting recognition) owing to their high predictive power and their relatively inexpensive computational cost (Denisko & Hoffman, 2018).

Random forests are a fit strategy in our case study for several reasons, since they (a) deal efficiently with correlated predictors (an important concern when dealing with morphological features), (b) naturally handle both categorical (e.g. material of which the panpipe is made of) and numeric (e.g. the number of tubes forming an instrument) variables and (c) allow for a natural solution to the problem of class imbalance. We consider an implementation where the suitability of each partition proposed by each decision tree is subject to a statistical test of association. In this manner, we build classifiers that avoid overfitting by latching only on sufficiently well attested regularities in the data, potentially revealing meaningful associations between traits and cultural history.

**Figure 3.**
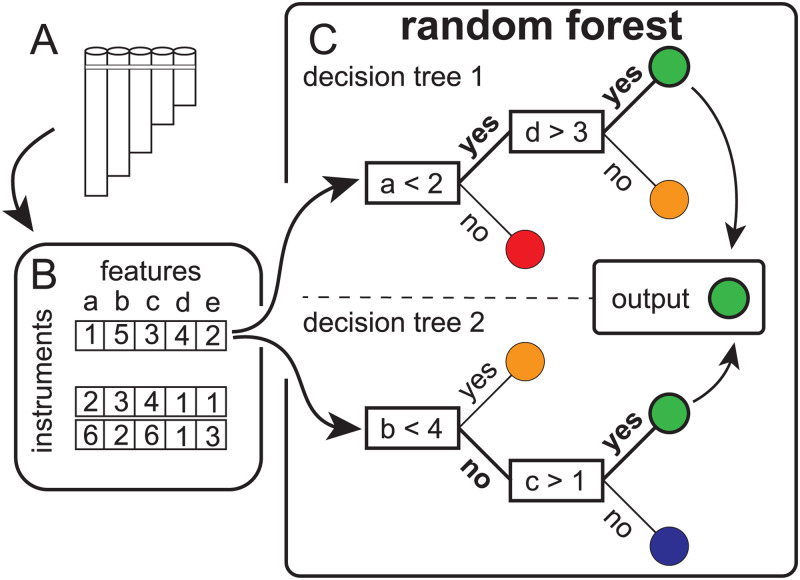
Decision trees vote for class outcome in a random forest example. Panpipe features were mostly obtained from collections with online databases (a) and collated into a matrix (b); each instrument is assessed by a set of decision trees formed by different decision points (rectangles) that end in a leaf belonging to a class (coloured circles, representing provenance). A random forest combines votes from its decision trees and produces a final class prediction, in this case the green area class (c). Figure after Denisko and Hoffman (2018).

Random forests are ensembles of decision trees, in this case trained on subsets of panpipes and features (Figure 3b). In the implementation used here (which follows the outline suggested by Strobl, Boulesteix, Zeileis, and Hothorn (2007), aimed at reducing the bias in classification), each decision tree is grown based on five randomly sampled features and 147 panpipes (which corresponds to a fraction of *e*^−1^ of all panpipes). The set of 147 panpipes is sampled with replacement from the original complete set and in such a manner that the probability of sampling any given instrument is inversely proportional to the total number of instruments associated with the corresponding region. In this manner we correct for the imbalance in the total number of panpipes across regions (Janitza & Hornung, 2018). Each individual decision tree in the ensemble is built by producing a binary partition of the data recursively (Figure 3c). In each iteration, standard association tests are performed between the variable that labels the regions and each of the individual traits. The trait yielding the strongest (and statistically significant) association is chosen for inducing the binary partition that produces the strongest distinction between regions according to the traits.

## Results

### Overall structure of panpipes

Of the 401 instruments in the dataset, there are 252 unique instrument descriptions. In comparison, 55% of all random permutations of the data give rise to sets where each observation is unique. Even though the features we use to characterize panpipes capture much of their complexity and their diversity, there are enough regularities among the instruments, resulting in combinations of features re-occurring in the dataset. This illustrates that, in high-dimensional settings, achieving a perfect classification (in this case determining the provenance of a panpipe given its traits) can be rather trivial. The biggest challenge is then to infer a classification that provides insights into the processes giving rise to the differences in the panpipes across provenances.

**Figure 4.**
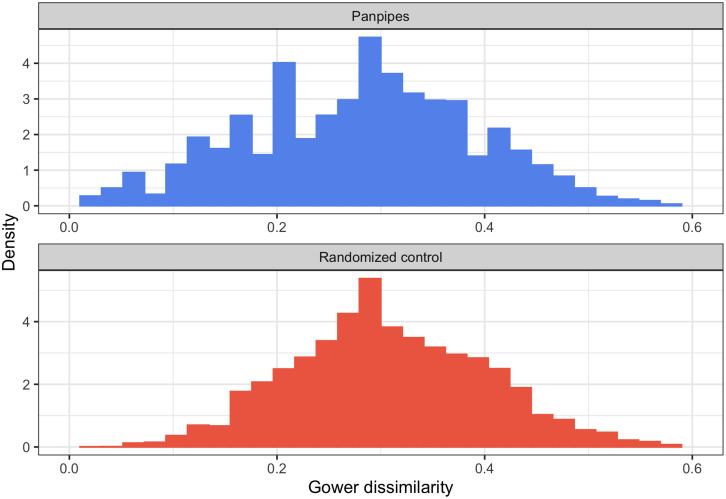
Gower dissimilarities in the panpipe dataset (top panel) and the average across *N* = 1000 permuted datasets (bottom panel).

The comparison between the distribution of Gower dissimilarities in the data and the average in the randomized baselines reveals modest differences consisting mainly of a more spiked distribution of panpipe dissimilarities (see Figure 4). This is expected given the smaller number of unique instruments in the empirical data in contrast to the randomized controls. Overall, both empirical and control distributions display a main unimodal component (i.e. most instruments are apart from each other around an average value). This rules out extreme situations where panpipes are concentrated in distant corners of the trait space. This would correspond to a case where each pair of panpipes is either very similar or very different systematically across traits.

**Figure 5.**
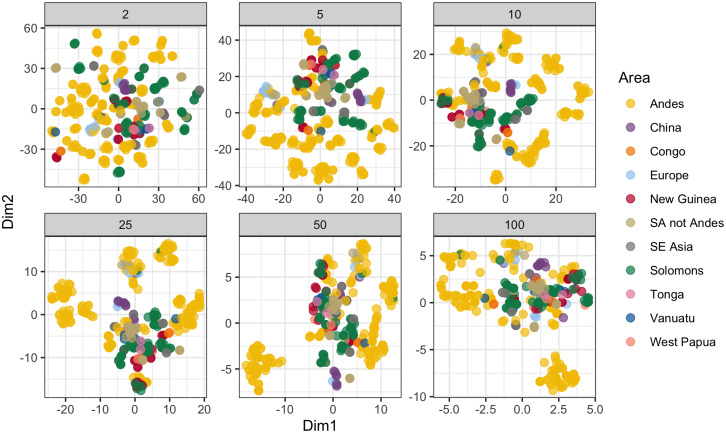
*t*-Distributed stochastic neighbour embedding (*t*-SNE) projection of the random forest data. The different panels represent perplexity values from 2 to 100. The spread of the Andes panpipes (yellow points) relates to a large diversity of instruments and features. The clustering of areas (as represented by colours) shows that the panpipes reflect their provenance.

Crucially, differences between panpipes do align with their provenance, as revealed in the *t*-SNE plots in Figure 5. Of note are the spread of the Andes data points, indicating a larger diversity in features/kinds of instruments.

### Patterns in provenance mismatches

Once we have established that panpipe traits display a non-trivial association with their provenance, we set out to evaluate a specific historical pattern of contact – namely the putative contact between the Pacific and South America. For this purpose we group the individual provenances of the panpipes into four wide regions: Andes, Melanesia, Amazon and Old World. In addition, we remove the variable ‘material’ from the pool of traits since it might lead to preservation bias: ceramic panpipes preserve longer than their organic counterparts, therefore only panpipes made of bamboo and reed are analysed. Features restricted to single regions, although diagnostic, are irrelevant when looking for patterns of diffusion or convergence; therefore only traits appearing in more than one region are analysed.

The random forests classifier is able to efficiently assign panpipes to their regions of origin, as attested by the confusion matrix in Table 1. Crucially, for the purpose of determining contact events between regions, we focus our attention on the few misclassified cases. In principle, misclassified instruments might result from various factors, including the presumed high likelihood of independent developments of the same artefact, as proposed by Izikowitz (1935). However, given the rich dataset under analysis and the overall excellent performance of the classifier, misclassification events might indicate the persistence of cultural practices through contact between regions.

**Table 1.** Confusion matrix and basic statistics resulting from the random forests classification. Rows indicate known origin and columns indicate predictions.

	Andes	Melanesia	Old World	SA not Andes	Recall
Andes	1288	3	5	1	0.93
Melanesia	3	877	1	13	0.84
Old World	0	1	39	0	0.98
SA not Andes	0	1	1	29	0.94
Precision	0.98	0.95	0.85	0.67	—

Table 1 shows that the precision (or positive predictive value) of the Andes is very high because 128 of 131 panpipes are successfully assigned to their provenance (three are falsely assigned to Melanesia). Similarly, the recall (or sensitivity) is 0.93 because 128 panpipes are successfully assigned to their provenance, but nine are wrongly assigned to other areas. The lowest values of precision and recall are those related to the 13 panpipes from Melanesia that are erroneously assigned as originating in the ‘SA not Andes’ region: MIM1855 is from Papua New Guinea; EMBVIId12a–d were made by the Kwaio people of central Malaita Island; MQB71.1970.101.1–4 and MQB71.1970.101.34 were made by the ‘Are'are of southern Malaita Island (Solomons Islands); MQB71.1890.63.7 is from the Solomon Islands (details unknown); MQB71.1930.29.439 is from Vanuatu; and MQB72.56.750.1 was collected in Tonga – panpipes are unusual in Polynesia, but reported for Tonga, probably introduced from neighbouring Fiji (Kaeppler, 1974). A closer look at the 13 instruments reveals that they include mostly plain (or basic) features such as: only one row of tubes, decreasing pattern of tube size, lack of splints in their ligature construction and simple knots. The Tongan panpipe is wrapped in a cloth, thus obscuring many relevant features that had to be left as unknown and probably are involved in the missclassification. The combination of such ‘generic’ features is found in many instruments of the ‘SA not Andes’ category.

## Discussion

The growing field of cultural evolution studies of music (Savage, 2019) could be expanded to include studies of musical instruments. Phylogenetic analyses of Baltic psaltery (Tëmkin, 2004) and cornets (Tëmkin & Eldredge, 2007), and shape analysis of violins (Chitwood, 2014) have provided insights into transmission dynamics and routes of diffusion. This study departs from previous work in the greater geographical and temporal scope, the relatively simple nature of the panpipe as opposed to the complexity of the psaltery, violins and cornets, and the methods being used for the analysis of the data. Random forests enabled a classification of instruments using their physical features without a phylogenetic framework. Our category scheme (using countries and roughly defined areas) is conservative and necessary for such an exhaustive study. This study contributes a novel approach to organological analyses. Although obvious, it should be noted that biases such as collection effort, the availability of certain types of instruments and the overall durability of different materials can contribute to a skewed perspective. This needs to be accounted for in future studies of panpipes.

Native South American aerophones (wind instruments) have probably the largest number and greatest diversity in the world (Olsen, 2004), as reflected in their ethnological (Izikowitz, 1935) and archaeological (Hickmann, 2008; Pérez de Arce & Gili, 2013) record. Such remarkable diversity of aerophones mirrors the high cultural, linguistic and genetic diversity in the Americas (Nettle, 1999; Nichols, 1990; Tarazona-Santos et al., 2001). Panpipes are the prime case of aerophone diversity in South America, even though this fact is rarely mentioned in the literature, if at all. The data collected and analysed here provide a preliminary view of the global richness (Figure 1) and diversity (Figure 5) of panpipes.

Random forests has a high predictive performance and other qualities that make it an attractive method for many fields, including cultural evolution. As with most classifiers, random forests may be affected by an imbalanced training dataset (in which some classes are much smaller than others). Random forests are constructed to minimize the overall error rate and therefore focus particularly on the prediction accuracy of the majority class, which often results in poor accuracy for the minority class (Chen, Liaw, & Breiman., 2004). To avoid issues related to sample size imbalance, we relied on the methods proposed by Strobl, Boulesteix, Zeileis, and Hothorn (2007) and Janitza and Hornung (2018). The panpipes of Melanesia and those in the ‘SA not Andes’ category share many features linked to a plain or basic design and do not represent a ‘breakthrough’ that would strongly imply some sort of diffusion. These plain features may readily be explained by chance, material availability or convergence. A meaningful comparison is challenging, partly because the time dimension is subject to a strong preservation bias: the archaeological record of panpipes of South America is impressive, but subject to a strong material bias (clay and rock panpipes subsist much longer than their organic counterparts). Regarding music itself, the similarities that von Hornbostel (1911) found in the pitches and scales of Oceanian and Amazonian instruments have also been discussed, with some authors supporting this as the definite proof of Trans-Pacific diffusion (e.g. Sachs, 1940; Campbell, 1960), whereas others interpreted this as the deep roots of Chinese music standards in all music (e.g. Fox-Strangways, 1929). Jones has argued in a series of papers (e.g. Jones, 1980, 1981) that the Equiheptatonic scale of the panpipes from Africa, Oceania, Indonesia and Peru is relevant to cultural diffusion studies. Sachs (1940) also discussed the influence of the Chinese tuning into the ‘twelve lü’, which involves two series of fifths complementing each other. This tuning, he argues, influences the dual nature of panpipes, such as those with two wings or those composed of two parts tied together with a lace (e.g. the Karenni and Guna instruments).

We conclude that panpipe features (and those of other musical instruments) are relevant to the growing field of cultural evolution of music and can be used to trace their provenance. Patterns of confusion in the random forests confusion matrix signal either potential diffusion events or cases of design convergence. Future analyses limiting the scope to a smaller area (e.g. a single continent or region) and selecting entries with finer-grained ethnological details (society, community) would probably provide potential diffusion routes that could be targeted to gain a better understanding. Expansion of the extensive database presented here should concentrate on South American panpipes outside the Andes. This preservation bias could be compensated for by the incorporation of iconography: figurines made from clay depict panpipes made from organic materials and demonstrate that they were also common in ancient South American cultures, such as Nasca and Moche.

## Data Availability

Geographic information, descriptions, and photos of most instruments can be found through the following urls: http://collections.quaibranly.fr (database of Musée du quai Branly) and https://mimo-international.com/MIMO/ (Musical instrument museums online database). Institutional information and other background data is detailed in Supplementary File 1. The matrix is available in csv format as Supplementary File 2. A Markdown file with code instructions for the random forest analysis and additional analyses is provided as Supplementary File 3.

## Supplementary material

For supplementary material accompanying this paper visit http://dx.doi.org/10.1017/ehs.2020.15. click here to view supplementary material
