# Supplementary material for: Panpipes as units of cultural analysis and dispersal
Source: Evol Hum Sci. 2020 May 21;2:e17. doi: 10.1017/ehs.2020.15 (PMC10427469; doi:10.1017/ehs.2020.15)
Supplement: Supplementary file 1 [file ehssup.zip › S2513843X20000158sup001.pdf]

Code for Aguirre, Blasi & Sanchez 2020

Damian E. Blasi

2/5/2020

Load libraries and set options

Loading and visualizing data

```
pan<-read.csv("/Users/damian/Desktop/Projects/Pan flutes/Final version/SuppFile2.csv",sep=",",
              stringsAsFactors = F,header=T,skip=1)
str(pan)
```

```
## 'data.frame':    401 obs. of  26 variables:
##  $ Collection.code                              : chr  "Elegant_Chiaohsiao_Tangdynasty" "Elegant_Shaol
##  $ Ethnological.group                           : chr  "China_Tang" "China_Tang" "Timor indet" "Timor
##  $ Area_fine                                    : chr  "China" "China" "SE_Asia" "SE_Asia" ...
##  $ Area_coarse                                  : chr  "Asia" "Asia" "Asia" "Asia" ...
##  $ Area_coarse_time                             : chr  "Asia" "Asia" "Asia" "Asia" ...
##  $ Dated..archaeological..ethnological..modern.: chr  "618?907" "618?907" "1888" "1888" ...
##  $ Size                                         : int  1 1 1 1 1 1 1 1 1 1 ...
##  $ Wider.than.high                              : chr  "1" "1" "0" "0" ...
##  $ Material                                     : int  2 2 2 2 2 2 2 2 2 2 ...
##  $ Raft.or.bundle                               : int  0 0 0 0 0 0 0 0 0 0 ...
##  $ One.or.two.rows                              : int  0 0 0 0 0 0 1 1 0 1 ...
##  $ Parts                                        : int  0 0 0 0 0 0 0 0 0 0 ...
##  $ No.pipes.S1                                  : chr  "24" "12" "10" "9" ...
##  $ Stopped.or.open                              : int  0 0 0 0 0 0 0 0 0 0 ...
##  $ Seriation.of.pipes                           : chr  "0" "0" "0" "0" ...
##  $ Ligature.material                            : chr  "4" "4" "3" "3" ...
##  $ Thread.ligature.knot                         : chr  "-" "-" "0" "0" ...
##  $ Splint.arrangement                           : chr  "4" "4" "4" "4" ...
##  $ Tubes.cut.below.node                         : chr  "0" "0" "0" "0" ...
##  $ Indentation.at.distal.end                    : chr  "0" "0" "2" "2" ...
##  $ Indentation.proximal.end                     : chr  "0" "0" "3" "3" ...
##  $ Distal.profile                               : chr  "0" "0" "1" "1" ...
##  $ cubical.tubes                                : chr  "0" "0" "0" "0" ...
##  $ painting                                     : chr  "0" "0" "0" "0" ...
##  $ carving.model                                : int  0 0 0 0 0 0 0 0 0 0 ...
##  $ textile                                      : int  0 0 0 0 0 0 2 0 0 0 ...
```

```
table(pan$Area_fine)
```

```
##
##       Andes        China        Congo       Europe    NewGuinea      Papuans
##         225           12            5           14           20            3
## SA_notAndes      SE_Asia     Solomons        Tonga      Vanuatu
##          31           10           72            2            7
```

```
table(pan$Area_coarse)
```

```
##
##      Africa        Andes         Asia       Europe    Melanesia SA_notAndes
##           5          225           22           14          104          31
```

Normalize NAs

```
pan<-as.data.frame(mapply(function(x)
  ifelse(x %in% c("?","-"),NA,x),pan))
```

We recode number of pipes to coincide with its four quartiles

```
pan[["No.pipes.S1"]]<-sapply(as.numeric(pan[["No.pipes.S1"]]),function(x)
  ifelse(is.na(x),NA,
          ifelse(x<=12,0,
           ifelse(x<=26,1,
            ifelse(x<=28,2,
             3)))))
```

Check overall missingness

```
nas<-apply(pan[,c(7:ncol(pan))],2,function(x) sum(is.na(x))/length(x))
fmax<-apply(pan[,c(7:ncol(pan))],2,function(x) max(table(x))/length(x[!is.na(x)]))
```

Plot fraction of missing data per feature

```
ggplot(data.frame(missing=nas,
                  id=sapply(names(nas),function(x) x),
                  threshold=sapply(nas,function(x) x>0.25)),
       aes(x=id,y=missing,color=threshold))+
  geom_point()+
  xlab("")+
  ylab("Fraction of missing data")+
  coord_flip()+
  theme(legend.position = "null")
```

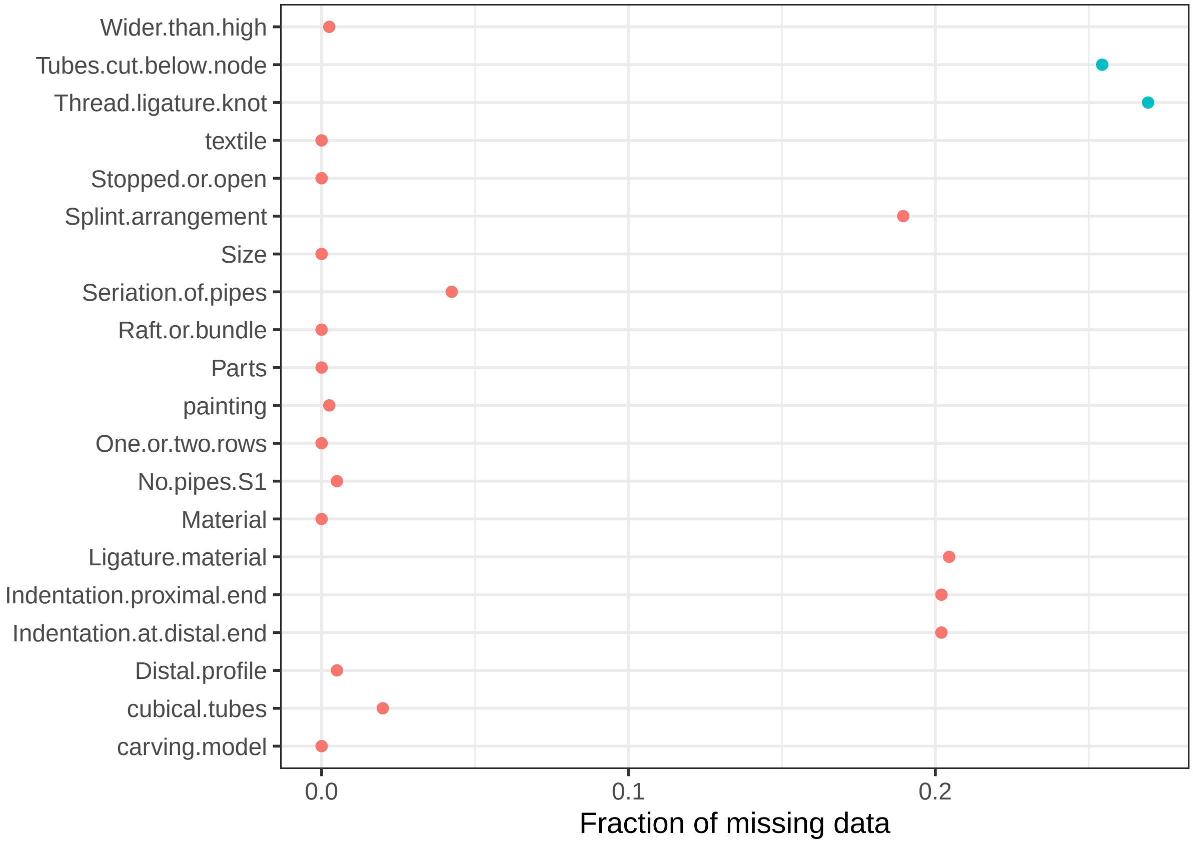

Plot frequency of most frequent level per feature

```
ggplot(data.frame(freqmax=fmax,
                  id=sapply(names(fmax),function(x) x),
                  threshold=sapply(fmax,function(x) x>0.9)),
       aes(x=id,y=freqmax,color=threshold))+
  geom_point()+
  xlab("")+
  ylab("Frequency of the most frequent value")+
  coord_flip()+
  theme(legend.position = "null")
```

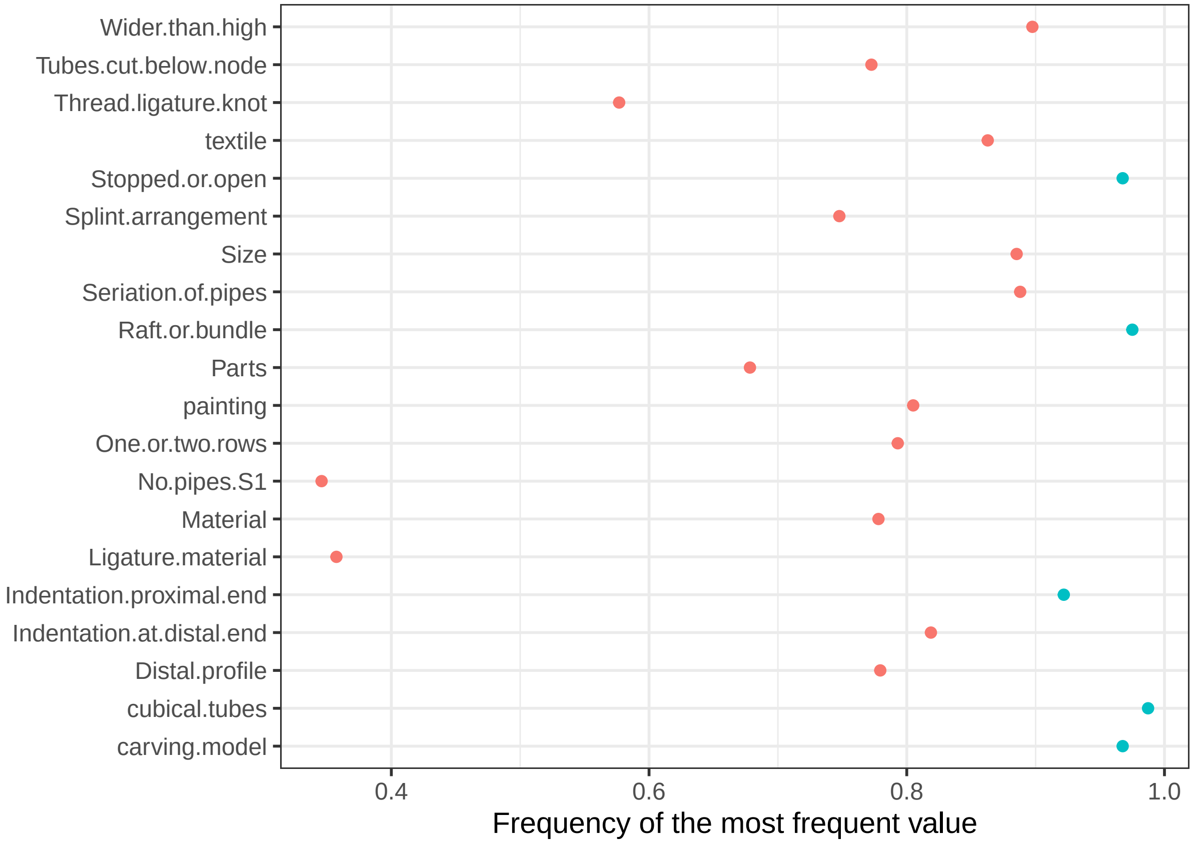

There are some features with a strong tendency of having the same coded value.

Plot nominal associations to assess the colinearity between traits

```
pan_gk<-GKtauDataframe(pan[,c(7:ncol(pan))])
assoc_feat<-(adply(pan_gk,1:2,function(x) ifelse(x>0.5,x,0)) %>% subset((V1>0)&(X1!=X2)))
print(assoc_feat)
```

```
##                             X1                         X2    V1
## 50           Ligature.material                   Material 0.818
## 51        Thread.ligature.knot                   Material 0.563
## 52          Splint.arrangement                   Material 0.826
## 53        Tubes.cut.below.node                   Material 0.702
## 54   Indentation.at.distal.end                   Material 0.827
## 55    Indentation.proximal.end                   Material 0.826
## 56              Distal.profile                   Material 0.748
## 58                    painting                   Material 0.741
## 68             Stopped.or.open             Raft.or.bundle 0.614
## 144             Raft.or.bundle            Stopped.or.open 0.614
## 210          Ligature.material       Thread.ligature.knot 0.501
## 230          Ligature.material         Splint.arrangement 0.743
## 263                   Material  Indentation.at.distal.end 0.529
## 270          Ligature.material  Indentation.at.distal.end 0.545
## 272         Splint.arrangement  Indentation.at.distal.end 0.533
## 275   Indentation.proximal.end  Indentation.at.distal.end 0.540
## 283                   Material   Indentation.proximal.end 0.717
```

```
## 290          Ligature.material  Indentation.proximal.end 0.759
## 291       Thread.ligature.knot  Indentation.proximal.end 0.504
## 292         Splint.arrangement  Indentation.proximal.end 0.664
## 293       Tubes.cut.below.node  Indentation.proximal.end 0.522
## 294 Indentation.at.distal.end  Indentation.proximal.end 0.724
## 296             Distal.profile  Indentation.proximal.end 0.571
## 298                   painting  Indentation.proximal.end 0.603
## 303                   Material            Distal.profile 0.712
## 310          Ligature.material            Distal.profile 0.691
## 311       Thread.ligature.knot            Distal.profile 0.529
## 312         Splint.arrangement            Distal.profile 0.669
## 313       Tubes.cut.below.node            Distal.profile 0.512
## 314 Indentation.at.distal.end            Distal.profile 0.637
## 315  Indentation.proximal.end            Distal.profile 0.636
## 318                   painting            Distal.profile 0.693
## 323                   Material             cubical.tubes 0.749
## 343                   Material                  painting 0.694
## 350          Ligature.material                  painting 0.681
## 352         Splint.arrangement                  painting 0.694
## 354 Indentation.at.distal.end                  painting 0.640
## 355  Indentation.proximal.end                  painting 0.641
## 356             Distal.profile                  painting 0.671
```

There are 39 dependencies where one variable predicts the other with a precision of over 50%, covering 12 unique variables. This entails that colinearity will play an important role in our results.

Overall structure in panpipe data

We attempt at determining the provenance group where a flute comes from based on its morphological features. We consider how unique trait combinations are

```
pan_traits<-colnames(pan[,c(7:ncol(pan))])
unique_pan<-ddply(pan[,c("Ethnological.group",pan_traits)],pan_traits,
                  function(x) data.frame(l=length(unique(x$Ethnological.group)),
                                       e=paste0(unique(x$Ethnological.group),collapse = "|")))
```

There are 192 instruments that are found only within one provenance. The rest have the following distribution of provenances:

```
unique_pan$e[unique_pan$l>1]
```

```
##  [1] Kwaio|Papua
##  [2] Kwaio|Areare
##  [3] Ecuador|Peru
##  [4] Ecuador|Peru
##  [5] Peru|Paracas
##  [6] English|Peru|Yaguas
##  [7] Lombards|English
##  [8] Peru|Aymara
##  [9] Peru|Bolivia|Aymara
## [10] Bolivia|Aymara
## [11] Ashaninka_Campa|Solomons
```

```
## [12] Wayana-Roucouyenne|Tupi|Nambikwara|Waura (Wauja)|Wayampi
## [13] Aymara|Peru
## [14] Nasioi|Papua
## [15] Colombia|Peru
## [16] Tupi|Yaguas
## [17] Waura (Wauja)|Ikpeng_Txikao
## [18] Urus|Aymara
## [19] Urus|Bolivia
## [20] Urus|Bolivia
## [21] Peru|Bolivia
## 66 Levels: China_Tang Timor indet Paracas Quechua Peru Pachacamac ... Karen
```

We compare this against 500 randomized versions of the pan flutes

```
nsim=500

baseline_unique<-function(DATA) {
  DATA<-as.data.frame(lapply(DATA,function(x) sample(x)))
  DATA$Ethnological.group<-DATA$Ethnological.group
  unique_rand<-ddply(DATA[,c("Ethnological.group",pan_traits)],pan_traits,
               function(x) data.frame(l=length(unique(x$Ethnological.group)),
                                        e=paste0(unique(x$Ethnological.group),collapse = "|")))
  return(sum(unique_rand$l==1))}

comparison_unique<-rdply(nsim,function(x) baseline_unique(pan[,c("Ethnological.group",pan_traits)]))
```

Most randomizations (0.19) yield 401 distinct instruments. Now we move to compare the distribution of Gower dissimilarities between instruments - both in the actual dataset and the randomized control.

```
nsim=500

baseline_comp<-function(DATA) {
  D<-as.data.frame(lapply(DATA,function(x) sample(x)))
  D$Ethnological.group<-DATA$Ethnological.group
  unique_rand<-ddply(D[,c("Ethnological.group",pan_traits)],pan_traits,
               function(x) data.frame(l=length(unique(x$Ethnological.group)),
                                        e=paste0(unique(x$Ethnological.group),collapse = "|")))
  return(as.vector(cluster::daisy(D))) }

comparison_random<-rdply(nsim,function(x) baseline_comp(pan[,c("Ethnological.group",pan_traits)]))
comparison_random<-data.frame(d=as.vector(as.matrix(comparison_random[,c(2:ncol(comparison_random))])),
                              type="random",stringsAsFactors = F)

distances_empirical<-cluster::daisy(pan[,pan_traits])
comparison_empirical<-data.frame(d=as.vector(distances_empirical),type="empirical",stringsAsFactors = F

distances<-rbind(comparison_random,comparison_empirical)
```

We plot the distribution of dissimilarities

```
ggplot(distances,aes(x=d,y=..density..,group=type,fill=type,color=type))+
  geom_histogram(bins=15)+
  scale_x_continuous(limits=c(0,0.6))+
```

```
  facet_wrap(~type,scales = "free",nrow=2,
             labeller = as_labeller(c("empirical"="Panpipes","random"="Randomized control")))+
  labs(x="Gower dissimilarity",y="Density")+
  theme(legend.position = "none")+
    scale_color_manual(values=c("cornflowerblue","coral2"))+
  scale_fill_manual(values=c("cornflowerblue","coral2"))
```

```
## Warning: Removed 53369 rows containing non-finite values (stat_bin).

## Warning: Removed 4 rows containing missing values (geom_bar).
```

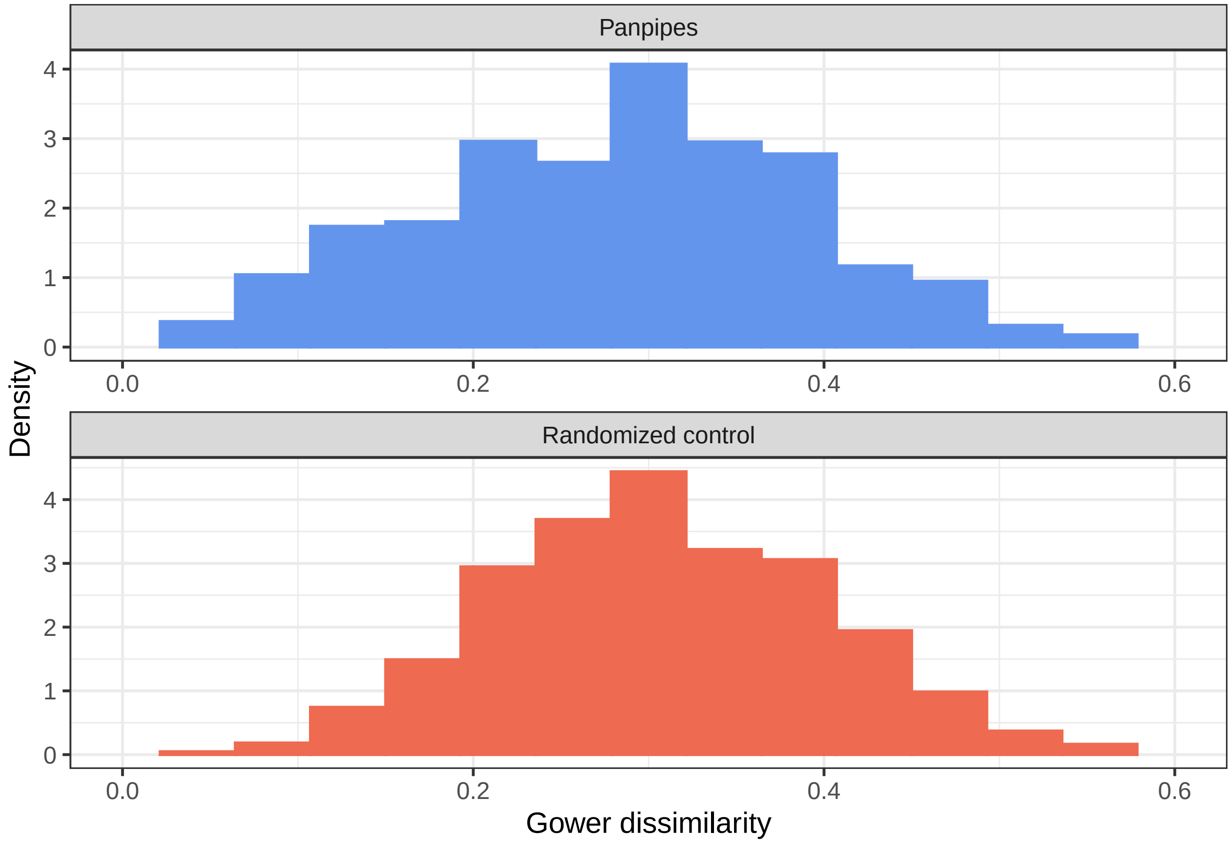


```
ggsave("panpipes_distances.tiff",dpi=300)
```

```
## Saving 6.5 x 4.5 in image

## Warning: Removed 53369 rows containing non-finite values (stat_bin).

## Warning: Removed 4 rows containing missing values (geom_bar).
```

Now we evaluate how do panpipes cluster in the trait space bty appealing to a t-SNE algorithm applied to the Gower dissimilarities between panpipes. Notice that we consider a range of perplexity values (a free parameter in the t-SNE algorithm that relates to the number of effective neighbors expected for each observation.)

```
set.seed(26111985)

perplexities<-c(2,5,10,25,50,100)
plots_tsne<-vector("list",length=length(perplexities))

for(ppx in 1:length(perplexities)) {
  tsne_p <- Rtsne(distances_empirical, dims = 2,
              perplexity=perplexities[ppx], verbose=FALSE, max_iter = 500,
              check_duplicates=F)
  if(ppx==1) tsne<-data.frame(Dim1=tsne_p$Y[,1],
                              Dim2=tsne_p$Y[,2],
                              Area=pan$Area_fine,
                              Perplexity=perplexities[ppx])
  else{tsne<-rbind(tsne,data.frame(Dim1=tsne_p$Y[,1],
                              Dim2=tsne_p$Y[,2],
                              Area=pan$Area_fine,
                              Perplexity=perplexities[ppx]))}}

  tsne$Macro<-sapply(as.character(tsne$Area),
                   function(x) ifelse(x %in% c("Africa","Europe","China","SE_Asia"),"Old World",
                                      ifelse(x %in% c("SA_notAndes","Andes"),"South America","Pacific")

   tsne$Area<-sapply(as.character(tsne$Area),
                    function(x) ifelse(x=="NewGuinea","New Guinea",
                                       ifelse(x=="Papuans","West Papua",
                                              ifelse(x=="SE_Asia","SE Asia",
                                                     ifelse(x=="SA_notAndes",
                                                     "SA not Andes",x)))))
  cvxhull <- tsne %>%
  group_by(Macro) %>%
  slice(chull(Dim1,Dim2))

  ## Plotting
  ggplot(tsne,aes(x=Dim1,y=Dim2,color=Area,label=Area))+
  scale_color_manual(values=as.vector(kelly(13)[c(3:13)]))+
  geom_point(alpha=0.7,size=2.5)+
    facet_wrap(~Perplexity,scales = "free")
```

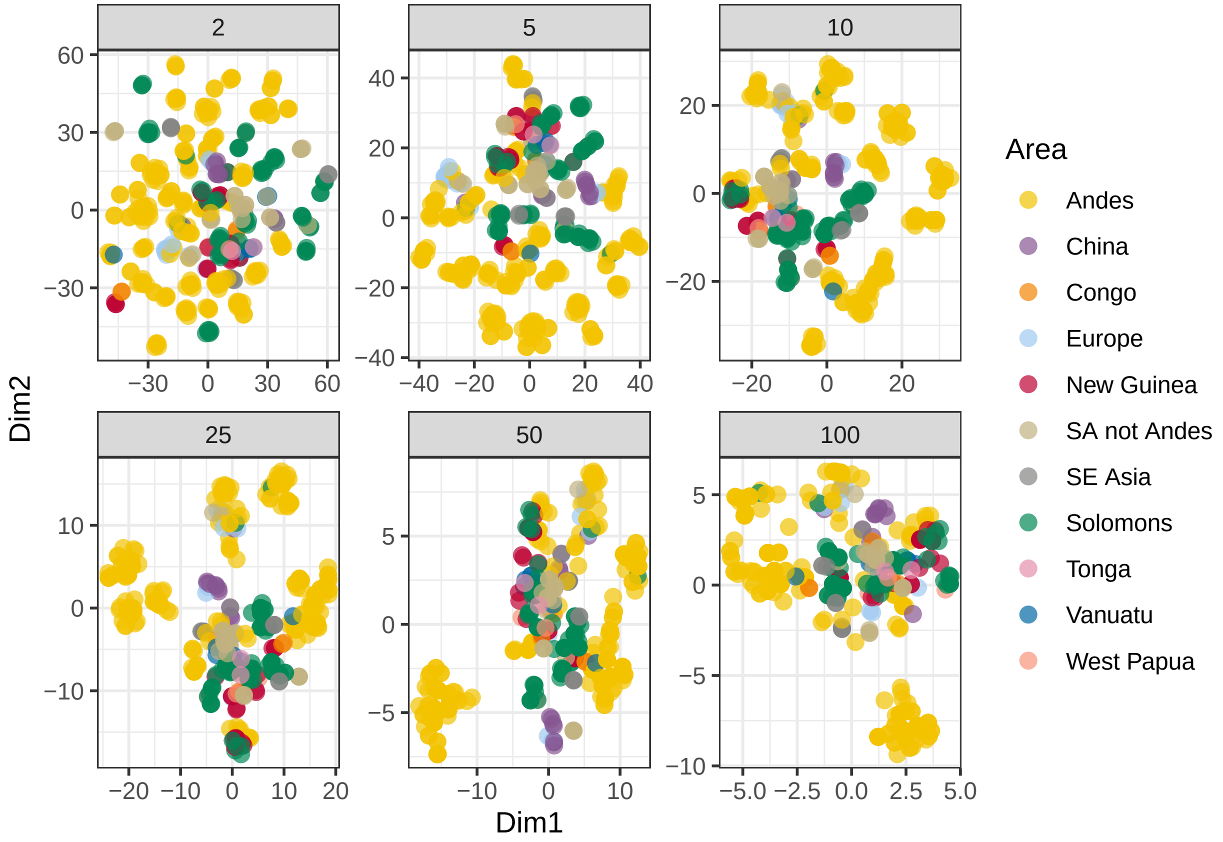


```
ggsave("tsne_panpipes.tiff",width=7.5,dpi=300)
```

```
## Saving 7.5 x 4.5 in image
```

Traceability of provenance given traits

Now we focus on potential indicators of cultural transmission. First we put together the Old World (for data size considerations)

```
pan$Region<-sapply(as.character(pan$Area_coarse),function(x)
  ifelse(x %in% c("Africa","Asia","Europe"),"Old World",x))
table(pan$Region)
```

```
##
##        Andes   Melanesia   Old World SA_notAndes
##          225         104          41          31
```

We remove trait levels that are present in a single region. We code NA for such levels.

```
pan_redux<-pan[,c(7:ncol(pan))]
for(i in 1:(ncol(pan_redux)-1)){
tab<-table(pan_redux[,c("Region",colnames(pan_redux)[i])])
check<-apply(tab,2,function(x) ifelse(sum(x!=0)==1,"fix","ok"))
```

```
pan_redux[,colnames(pan_redux)[i]]<-sapply(pan_redux[,colnames(pan_redux)[i]],
                                           function(x) ifelse(x %in% names(check)[check=="fix"],NA,as.c
pan_redux[,colnames(pan_redux)[i]]<-as.factor(pan_redux[,colnames(pan_redux)[i]])}
pan_redux<-pan_redux[,sapply(pan_redux, function(x)!all(is.na(x)))]
pan_redux$Region<-as.factor(pan_redux$Region)
```

We focus only on pan pipes produced out of vegetal sources, since those are the only ones present around the globe

```
print(summary(pan_redux$Region))
```

```
##      Andes   Melanesia   Old World SA_notAndes
##        225         104          41          31
```

```
pan_redux<-subset(pan_redux,Material==2)
print(summary(pan_redux$Region))
```

```
##      Andes   Melanesia   Old World SA_notAndes
##        137         104          40          31
```

```
pan_redux<-pan_redux[,colnames(pan_redux)!="Material"]
print(summary(pan_redux$Region))
```

```
##      Andes   Melanesia   Old World SA_notAndes
##        137         104          40          31
```

We train an efficient weighted random forest (or more precisely, a random conditional inference forest) to predict the region of origin of a pipe given its features

```
weights_pan<-sapply(pan_redux$Region,
                    function(x) nrow(pan_redux)/nrow(pan_redux[pan_redux$Region==x,]))
rf_redux<-cforest(Region~.,
                  controls=
                    cforest_control(ntree = 500,
                                    mtry=5,
                                    mincriterion=qnorm(0.9),
                                    fraction = 0.632,
                                    testtype ="Teststatistic",
                                    teststat="max",
                                    replace=TRUE,
                                    trace=F,
                                    savesplitstats=F,
                                    minsplit=20,
                                    minbucket=8),
                  data=pan_redux,
                  weights = weights_pan)
```

Produce the confusion matrix

```r
cmat_redux<-table(pan_redux$Region, predict(rf_redux))
print(cmat_redux)
```

```
##
##              Andes Melanesia Old World SA_notAndes
##   Andes        128         3         5           1
##   Melanesia      3        87         1          13
##   Old World      0         1        39           0
##   SA_notAndes    0         1         1          29
```

Statistics per region

```r
stats_region<-ldply(c(1:nrow(cmat_redux)),
                  function(x) data.frame(id=rownames(cmat_redux)[x],
                                         N=sum(cmat_redux[,x]),
                                         d=cmat_redux[x,x],
                                         A=sum(cmat_redux[x,]))) %>% transform(p=d/N,
                                                                      r=d/A)

stats_region
```

```
##            id   N   d   A         p         r
## 1       Andes 131 128 137 0.9770992 0.9343066
## 2   Melanesia  92  87 104 0.9456522 0.8365385
## 3   Old World  46  39  40 0.8478261 0.9750000
## 4 SA_notAndes  43  29  31 0.6744186 0.9354839
```

Obtain all the panpipes that are misclassified

```r
pan_redux$predReg<-predict(rf_redux)
missclass<-cbind(pan[rownames(pan_redux[pan_redux$Region!=pan_redux$predReg,]),],
                 data.frame(predReg=pan_redux$predReg[pan_redux$Region!=pan_redux$predReg]))
missclass
```

```
##       Collection.code Ethnological.group    Area_fine Area_coarse
## 7           EMVA12138            Paracas        Andes       Andes
## 8           EMVA16097            Paracas        Andes       Andes
## 15          EMVA40298         Pachacamac        Andes       Andes
## 25         EMVIId12a              Kwaio     Solomons   Melanesia
## 26         EMVIId12b              Kwaio     Solomons   Melanesia
## 27         EMVIId12c              Kwaio     Solomons   Melanesia
## 28         EMVIId12d              Kwaio     Solomons   Melanesia
## 51      MAEB15381.01            Paracas        Andes       Andes
## 63         MAEB15985     Tihuanaco-Huari        Andes       Andes
## 76           MIM1855              Papua    NewGuinea   Melanesia
## 82      MIM1979.028-03             Peru        Andes       Andes
## 83      MIM1979.028-04           Yaguas SA_notAndes SA_notAndes
## 90      MIM1984.036-06             Peru        Andes       Andes
## 91      MIM1984.036-07             Peru        Andes       Andes
## 92      MIM1984.036-08             Peru        Andes       Andes
## 113          MIM2169               Luba        Congo      Africa
```

```
## 132     MQB71.1890.63.7          Solomons    Solomons   Melanesia
## 143   MQB71.1930.29.439           Vanuatu     Vanuatu   Melanesia
## 151 MQB71.1934.188.1271             Papua   NewGuinea   Melanesia
## 153 MQB71.1934.188.1370       Bouganville   NewGuinea   Melanesia
## 168   MQB71.1937.0.104X           Vanuatu     Vanuatu   Melanesia
## 188  MQB71.1961.103.105          Solomons    Solomons   Melanesia
## 189    MQB71.1963.57.36      Ache_Guayaki SA_notAndes SA_notAndes
## 197    MQB71.1970.101.1            Areare    Solomons   Melanesia
## 208    MQB71.1970.101.2            Areare    Solomons   Melanesia
## 219    MQB71.1970.101.3            Areare    Solomons   Melanesia
## 224   MQB71.1970.101.34            Areare    Solomons   Melanesia
## 230    MQB71.1970.101.4            Areare    Solomons   Melanesia
## 265      MQB72.56.750.1           Tongans       Tonga   Melanesia
##       Area_coarse_time Dated..archaeological..ethnological..modern. Size
## 7    Andes precolonial                  800 BC - 100 BC (wikipedia)    1
## 8    Andes precolonial                  800 BC - 100 BC (wikipedia)    1
## 15   Andes precolonial                      200-1500 AD (wikipedia)    0
## 25           Melanesia                                 Ethnological    1
## 26           Melanesia                                 Ethnological    1
## 27           Melanesia                                 Ethnological    1
## 28           Melanesia                                 Ethnological    1
## 51   Andes precolonial                  800 BC - 100 BC (wikipedia)    1
## 63   Andes precolonial                      500-1000 AD (Wikipedia)    1
## 76           Melanesia                                 Ethnological    1
## 82  Andes postcolonial                                 Ethnological    1
## 83         SA_notAndes                                 Ethnological    1
## 90  Andes postcolonial                                 Ethnological    1
## 91  Andes postcolonial                                 Ethnological    1
## 92  Andes postcolonial                                 Ethnological    1
## 113             Africa                                 Ethnological    1
## 132          Melanesia                           late 19th century    1
## 143          Melanesia                                 Ethnological    1
## 151          Melanesia                                 Ethnological    1
## 153          Melanesia                                 Ethnological    1
## 168          Melanesia                                 Ethnological    1
## 188          Melanesia                                 Ethnological    1
## 189        SA_notAndes                                 Ethnological    1
## 197          Melanesia                                 Ethnological    2
## 208          Melanesia                                 Ethnological    1
## 219          Melanesia                                 Ethnological    1
## 224          Melanesia                                 Ethnological    1
## 230          Melanesia                                 Ethnological    1
## 265          Melanesia                            some centuries old    1
##     Wider.than.high Material Raft.or.bundle One.or.two.rows Parts No.pipes.S1
## 7                 0        2              0               1     0           1
## 8                 0        2              0               1     0           1
## 15                0        2              0               1     0           1
## 25                0        2              0               0     0           2
## 26                0        2              0               0     0           2
## 27                0        2              0               0     0           1
## 28                0        2              0               0     0           1
## 51                0        2              0               0     0           1
## 63                0        2              0               0     0           0
## 76                0        2              0               0     0           2
```

```
## 82                1        2             0              0     0           0
## 83                1        2             0              0     0           0
## 90                0        2             0              2     0           2
## 91                1        2             0              0     0           1
## 92                0        2             0              0     0           1
## 113               0        2             0              0     0           3
## 132               0        2             0              0     0           0
## 143               0        2             0              0     0           1
## 151               0        2             0              0     0           0
## 153               0        2             0              1     0           0
## 168               0        2             0              0     0           2
## 188               0        2             0              1     0           3
## 189               0        2             0              0     0           1
## 197               0        2             0              0     0           1
## 208               0        2             0              0     0           1
## 219               0        2             0              0     0           1
## 224               0        2             0              0     0           2
## 230               0        2             0              0     0           1
## 265               0        2             0              0     0           2
##     Stopped.or.open Seriation.of.pipes Ligature.material Thread.ligature.knot
## 7                 0                  0                 0                    4
## 8                 0                  0                 0                    4
## 15                0                  0                 0                    2
## 25                0                  0                 0                    3
## 26                0                  0                 0                 <NA>
## 27                0                  0                 0                    3
## 28                0                  0                 0                 <NA>
## 51                0                  0                 0                    0
## 63                0                  0                 3                    0
## 76                0                  0                 0                    3
## 82                0                  0                 1                    0
## 83                0                  0                 1                    0
## 90                0                  0                 2                    6
## 91                0                  2                 1                    0
## 92                0                  0                 3                    0
## 113               0                  0                 0                    0
## 132               0                  0                 1                    0
## 143               0               <NA>                 0                    0
## 151               0                  1                 0                    5
## 153               0                  0                 0                    5
## 168               0                  0                 2                    6
## 188               0                  0                 1                    0
## 189               0               <NA>                 2                    0
## 197               0                  0                 0                    3
## 208               0                  0                 0                    3
## 219               0                  0                 0                    3
## 224               0                  4                 0                    0
## 230               0                  0                 0                    3
## 265               0                  0                 0                 <NA>
##     Splint.arrangement Tubes.cut.below.node Indentation.at.distal.end
## 7                    4                    0                         1
## 8                    4                 <NA>                         1
## 15                   4                    0                         0
## 25                   4                    0                         0
```

```
## 26                   4                    0                         0
## 27                   4                    0                         0
## 28                   4                    0                         0
## 51                   4                    0                         0
## 63                   4                    0                         0
## 76                   4                    0                         0
## 82                   1                    0                         0
## 83                   1                    0                         0
## 90                   4                    1                         0
## 91                   1                    0                         0
## 92                   4                    0                         0
## 113                  4                    1                         0
## 132                  0                    0                         0
## 143                  4                    0                         2
## 151                  4                    1                         0
## 153                  4                    0                         0
## 168                  4                    0                         2
## 188                  0                    0                         1
## 189                  4                    0                         0
## 197                  4                    0                         0
## 208                  4                    0                         0
## 219                  4                    0                         0
## 224                  4                    0                         0
## 230                  4                    0                         0
## 265                  4                 <NA>                         0
##      Indentation.proximal.end Distal.profile cubical.tubes painting
## 7                           0              1             0        0
## 8                           0              1             0        0
## 15                          0              1             0        0
## 25                          0              1             0        0
## 26                          0              1             0        0
## 27                          0              1             0        0
## 28                          0              1             0        0
## 51                          0              1             0        0
## 63                          0              1             0        0
## 76                          0              1             0        0
## 82                          0              1             0        0
## 83                          0              1             0        0
## 90                          0              0             0        0
## 91                          1              1             0        0
## 92                          3              1             0        0
## 113                         0              1             0        0
## 132                         0              1             0        0
## 143                         0              1             0        0
## 151                         0              1             0        0
## 153                         0              1             0        0
## 168                         0              1             0        0
## 188                         0              1             0        0
## 189                         0              1             0        0
## 197                         0              1             0        0
## 208                         0              1             0        0
## 219                         0              1             0        0
## 224                         0              1             0        0
## 230                         0              1             0        0
```

```
## 265                        0              1             0        0
##      carving.model textile      Region     predReg
## 7                0       2       Andes   Melanesia
## 8                0       0       Andes   Melanesia
## 15               0       0       Andes   Melanesia
## 25               0       0   Melanesia SA_notAndes
## 26               0       0   Melanesia SA_notAndes
## 27               0       0   Melanesia SA_notAndes
## 28               0       0   Melanesia SA_notAndes
## 51               0       2       Andes SA_notAndes
## 63               0       2       Andes   Old World
## 76               0       0   Melanesia SA_notAndes
## 82               0       0       Andes   Old World
## 83               0       0 SA_notAndes   Old World
## 90               0       0       Andes   Old World
## 91               0       1       Andes   Old World
## 92               0       0       Andes   Old World
## 113              0       0   Old World   Melanesia
## 132              0       0   Melanesia SA_notAndes
## 143              0       0   Melanesia SA_notAndes
## 151              0       0   Melanesia   Old World
## 153              0       0   Melanesia       Andes
## 168              0       0   Melanesia       Andes
## 188              0       0   Melanesia       Andes
## 189              0       0 SA_notAndes   Melanesia
## 197              0       0   Melanesia SA_notAndes
## 208              0       0   Melanesia SA_notAndes
## 219              0       0   Melanesia SA_notAndes
## 224              0       0   Melanesia SA_notAndes
## 230              0       0   Melanesia SA_notAndes
## 265              0       0   Melanesia SA_notAndes
```
